# Effect of lipid emulsion on neuropsychiatric drug-induced toxicity: A narrative review

**DOI:** 10.1097/MD.0000000000037612

**Published:** 2024-03-15

**Authors:** Yeran Hwang, Ju-Tae Sohn

**Affiliations:** a Department of Anesthesiology and Pain Medicine, Gyeongsang National University College of Medicine, Gyeongsang National University Hospital, Jinju-si, Republic of Korea; b Institute of Health Sciences, Gyeongsang National University, Jinju-si, Republic of Korea.

**Keywords:** Glasgow Coma Scale, lipid emulsion, lipid solubility, lipid soluble, neuropsychiatric drugs, QTc, toxicity

## Abstract

Lipid emulsion has been shown to effectively relieve refractory cardiovascular collapse resulting from toxic levels of nonlocal anesthetics. The goal of this study was to examine the effect of lipid emulsions on neuropsychiatric drug-induced toxicity using relevant case reports of human patients, with a particular focus on the Glasgow Coma Scale (GCS) score and corrected QT interval, to analyze drugs that frequently require lipid emulsion treatment. The following keywords were used to retrieve relevant case reports from PubMed: “antidepressant or antipsychotic drug or amitriptyline or bupropion or citalopram or desipramine or dosulepin or dothiepin or doxepin or escitalopram or fluoxetine or haloperidol or olanzapine or phenothiazine or quetiapine or risperidone or trazodone” and “lipid emulsion or Intralipid.” Lipid emulsion treatment reversed the corrected QT interval prolongation and decreases in Glasgow Coma Scale scores caused by toxic doses of neuropsychiatric drugs, especially lipid-soluble drugs such as amitriptyline, trazodone, quetiapine, lamotrigine, and citalopram. The log *P* (octanol/water partition coefficient) of the group which required more than 3 lipid emulsion treatments was higher than that that of the group which required less than 3 lipid emulsion treatments. The main rationale to administer lipid emulsion as an adjuvant was as follows: hemodynamic depression intractable to supportive treatment (88.3%) > lipophilic drugs (8.3%) > suspected overdose or no spontaneous breathing (1.6%). Adjuvant lipid emulsion treatment contributed to the recovery of 98.30% of patients with neuropsychiatric drug-induced toxicity. However, further analyses using many case reports are needed to clarify the effects of lipid emulsion resuscitation.

## 1. Introduction

Currently, local anesthetic-induced systemic toxicity is treated with lipid emulsion, which is primarily used for parenteral nutrition.^[1]^ Supportive treatments, which are used to alleviate central nervous and cardiovascular symptoms caused by toxicity due to other substances (including neuropsychiatric drugs without a specific antidote), include charcoal, gastric lavage, fluid administration, sodium bicarbonate, inotropic agents, vasopressors, anti-arrhythmic drugs, and defibrillation.^[2,3]^ However, when supportive treatment does not improve the symptoms caused by neuropsychiatric drugs toxicity, lipid emulsion as adjuvant drug has been reported to be effective in ameliorating intractable central nervous system and cardiovascular system symptoms caused by toxic doses of antipsychotics and antidepressants.^[2–4]^ In 2008, Siriari et al^[5]^ reported a clinical case regarding lipid emulsion resuscitation of intractable cardiac arrest due to toxic doses of bupropion (an antidepressant) and lamotrigine (an anticonvulsant); this was the first successful lipid emulsion resuscitation for nonlocal anesthetic toxicity. Antidepressants, including amitriptyline and fluoxetine, and antipsychotics, such as quetiapine and haloperidol, produce corrected QT interval (QTc) prolongation, which leads to Torsade de Pointes, ventricular fibrillation, and cardiac arrest.^[6]^ SMOFlipid was reported to reverse the decreased Glasgow Coma Scale (GCS) score and QTc prolongation induced by clozapine (an atypical antipsychotic) toxicity.^[7]^ Intralipid increased the GCS scores of patients with acute toxicity caused by various nonlocal anesthetic drugs.^[8]^ In addition, the time required to recovery from sevoflurane and isoflurane anesthesia was shortened by lipid emulsion in rats, and appeared to be mediated by lipid emulsion-mediated reduction of sevoflurane and isoflurane concentrations.^[9]^ However, a systemic analysis of case reports regarding the effect of lipid emulsion on neuropsychiatric drug-induced toxicity, including antidepressants, antipsychotics, benzodiazepines (sedatives), and anticonvulsants, has not previously been reported. Moreover, a randomized controlled study regarding the effect of lipid emulsion on neuropsychiatric drug-induced toxicity in humans is impossible due to ethics concerns.^[10]^ Thus, in this review, we analyzed case reports on lipid emulsion treatment for neuropsychiatric drug-induced toxicity retrieved from PubMed until December 20, 2023. The goal of this review was to investigate the effect of lipid emulsion on symptoms caused by neuropsychiatric drug toxicity, with a particular focus on QTc prolongation, decreased GCS scores, and the lipophilicity of drugs that more frequently require lipid emulsion treatment.

## 2. Methods

Institutional review board approval was not needed because this was a narrative review using case reports.

### 2.1. Case search

**Figure 1.**
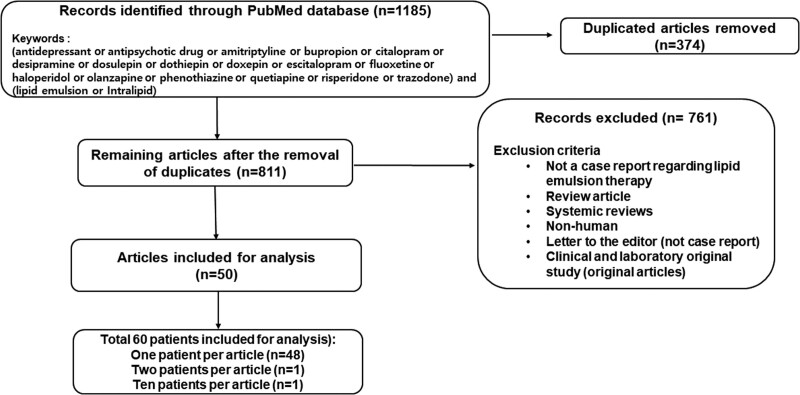
Flow chart for retrieving clinical case reports on lipid emulsion treatment as an adjuvant therapy for systemic neuropsychiatric (antidepressants, antipsychotics, benzodiazepines, and anticonvulsants) drug toxicity based on a PubMed keyword search. “n” indicates the number of articles.

Based on the Preferred Reporting Items for Systematic Reviews and Meta-Analyses guideline,^[11]^ the following terms were used to search for relevant case reports involving humans regarding the effect of lipid emulsion on drug toxicity caused by neuropsychiatric drugs until December 20, 2023: “antidepressant or antipsychotic drug or amitriptyline or bupropion or citalopram or desipramine or dosulepin or dothiepin or doxepin or escitalopram or fluoxetine or haloperidol or olanzapine or phenothiazine or quetiapine or risperidone or trazodone” and “lipid emulsion or Intralipid.” We retrieved a total of 1185 articles from PubMed (Fig. 1), which is Preferred Reporting Items for Systematic Reviews and Meta-Analyses flow diagram.

### 2.2. Eligibility criteria

After removing duplicate articles (n = 374), 811 articles remained. A further 761 articles were excluded for the following reasons: no case report regarding lipid emulsion therapy, review article, systemic review, nonhuman cases, letter to the editor (not case report), and/or an original clinical and laboratory research article (Fig. 1). Finally, 50 articles were included in the analysis. As 1 article contained 2 patients and another article contained 10 patients, the 50 articles included 60 patients (Fig. 1).

### 2.3. Data extraction

Pre- and post-lipid emulsion treatment QTc intervals and GCS scores were obtained from each case report based on measurements just before and after the administration of lipid emulsion, respectively. QTc intervals were calculated using the Bazett formula (QTc = QT/RR^1/2^).^[6]^ All other data, including age, sex, underlying diseases, neuropsychiatric drugs, dosage, lipid emulsion treatment information, improvement of symptoms, and outcomes, were obtained from the case reports. Log *P* (octanol/water partition coefficient) was obtained from PubChem.^[12]^

### 2.4. Statistical analysis

Normality tests for all data, which included log *P* of the drug, GCS score, and QTc, were performed using Shapiro–Wilk tests (Prism 5.0; GraphPad Software, San Diego, CA). The effect of lipid emulsion on GCS score was analyzed using Wilcoxon signed-rank tests. The effect of lipid emulsion on QTc prolongation was analyzed using paired Student’s *t* tests. The log *P* values of the groups which required more than or less than 3 lipid emulsion treatments were compared using the Mann–Whitney test. *P* values below .05 indicate statistical significance.

## 3. Results

### 3.1. Sex, age, underlying disease, and drugs

The numbers of male and female patients (total patients: 60) with systemic neuropsychiatric drug-induced toxicity who underwent lipid emulsion treatment as an adjuvant drug were 20 (33.33%) and 37 (61.66%), respectively (Table 1). The age distribution was as follows (Table 1): 20–29 years (16 patients, 26.6%), 10–19 years (10 patients, 16.6%), 30–39 years (10 patients, 16.6%), 50–59 years (9 patients, 15%), 40–49 years (8 patients, 13.3%), 60–69 years (3 patients, 5%), and <10 years (2 patients, 3.3%). The incidence of underlying diseases were as follows (Table 1): depression (18 patients, 30%), bipolar disorder (5 patients, 8.33%), schizophrenia (3 patients, 5%), and epilepsy (2 patients, 3.33%). Two patients did not have any underlying diseases. The drugs that caused toxicity, which were mainly neuropsychiatric drugs, were as follows (n = 39; Table 1): antidepressants including amitriptyline, nortriptyline, dosulepine (dothiepine), doxepine, citalopram, escitalopram, sertraline, fluoxetine, venlafaxine, bupropion, mirtazapine, and trazodone; antipsychotics including olanzapine, quetiapine, chlorpromazine, haloperidol, aripiprazole, amisulpride, zopiclone, and paliperidone; benzodiazepines including diazepam, clonazepam, bromazepam, and alprazolam; anticonvulsants including pregabalin, gabapentin, and lamotrigine; and other drugs including metoprolol, propranolol, cyclobenzapine, hydroxyzine, nifedipine, quinapril, aspirin, valsartan, ibuprofen, insulin, amoxicillin, and clonidine. The specific drugs which most frequently caused neuropsychiatric drug toxicity (total number of drugs including duplicates: 109) and required lipid emulsion treatment as an adjuvant drug were as follows (Table 1): amitriptyline (19.2%) > quetiapine (11%) > venlafaxine (6.4%) > citalopram or olanzapine (5.5%) > lamotrigine or bupropion (4.6%) > trazodone (3.7%) > dosulepin or sertraline or fluoxetine (2.8%) > zopiclone or escitalopram or cyclobenzaprine or diazepam or metoprolol or gabapentin (1.8%) > others (0.9%).

**Table 1:** Lipid emulsion treatment for toxicity caused by neuropsychiatric drugs (e.g., antidepressants, antipsychotics, benzodiazepines, and anticonvulsants).

Case no.	Sex	Age	Underlying disease	Drug	Dosage	Log *P*	Pre-LE QTc	Pre-LE GCS	Kind of LE	Post-LE QTc	Post-LE GCS	Improved symptoms	Outcome
1^[13]^	F	28	Depression	Amitriptyline	3.7 g	4.92	475 ms	N/A (coma)	20% LE	441 ms	WNL	CNS, CV	R
2^[14]^	F	77	Depression	Trazodone	4.5 g	2.68	540 ms	3	20% Intralipid	411 ms	10	CNS, CV	R
3^[15]^	F	15	N	Bupropion	1650–9000 mg	3.6	461 ms	11	20% Intralipid	N/A	N/A	CNS, CV	R
4^[16]^	N/A	25	Depression	Amitriptyline Quetiapine	5.5 g 10 g	4.92 2.81	650 ms	3	20% SMOFlipid	509 ms	N/A	CNS, CV Normothermia	R QTc Prolongation state (120%)
5^[17]^	F	19	N	Quetiapine Citalopram Bromazepam	6000 mg 400 mg 45 mg	2.81 3.76 2.05	570 ms	11	20 % Lipofundin MCT/LCT	370 ms	15	CNS, CV	R
6^[18]^	F	14	N/A	Bupropion Hydroxyzine Citalopram	9 g N/A N/A	3.6 2.36 3.76	527 ms	N/A	20% LE	N/A	N/A	CNS, CV	R
7^[19]^	M	21	Depression	Citalopram Clonazepam Olanzapine	11.6 g 5 mg 600 mg	3.76 2.41 3.0	487 ms	N/A	10% LE	N/A	N/A	CV	R
8^[20]^	F	42	Schizoaffective disorder	Quetiapine	24 g	2.81	467 ms	3	20% Intralipid	normal	N/A	CV	R
9^[21]^	F	34	N/A	Amitriptyline Citalopram	5600 mg 2400 mg	4.92 3.76	550 ms	3	20% Intralipid	N/A	N/A	CNS, CV	R
10^[22]^	F	13	N/A	Amitriptyline Nortriptyline	N/A	4.92 3.9	477 ms	N/A	20% LE	N/A	N/A	CNS, CV	R
11^[23]^	F	25	Anorexia Depression	Amitriptyline Fluoxetine Escitalopram Olanzapine Quetiapine Gabapentin	N/A	4.92 4.05 3.74 3.0 2.81 1.25	611 ms	N/A	20% LE	N/A	N/A	CV	R
12^[24]^	M	51	Depression IHD	Amitriptyline Quetiapine Citalopram Metoprolol Quinapril Aspirin	3250 mg N/A N/A N/A N/A N/A	4.92 2.81 3.76 2.15 .86 1.18	570 ms	N/A	20% LE	500 ms	N/A	CV	R
13^[25]^	F	36	N/A	Dothiepin	2250 mg	4.49	502 ms	N/A	20% Intralipid	N/A	N/A	CV	R
14^[26]^	F	36	Schizophrenia	Dosulepin	5.25 g	4.49	520 ms	10	20% Intralipid	506 ms	N/A	N/A	R
15^[5]^	F	17	Bipolar disorder	Bupropion Lamotrigine	4 g 7.95 g	3.6 2.57	485 ms	3	20% Intralipid	N/A	N/A	CV	CNS sequelae
16^[27]^	F	45	Anxiety Depression Hypertension	Haloperidol	5 g	4.3	545 ms	N/A	20% LE	385 ms	N/A	CV	R
17^[28]^	M	54	Anxiety Depression CAD CHF COPD type II DM	Trazodone	100 mg	2.68	539 ms	N/A	20% LE	485 ms	N/A	CV	R
18^[29]^	F	45	N/A	Amisulpride Diazepam Valsartan Aripiprazole Paliperidone	28 g 250 mg 2240 mg 45 mg 21 mg	1.06 2.82 1.499 5.30 1.8	547 ms	N/A	20% SMOFlipid	541 ms	N/A	CV	R
19^[30]^	M	36	Bipolar disorder	Lamotrigine	13.5 g	2.57	458 ms	N/A	20% LE	392 ms	N/A	CV	CNS sequelae
20^[31]^	F	25	N/A	Amitriptyline Propranolol Pregabalin	N/A	4.92 3.48 1.3	503 ms	3	20% Intralipid	444 ms	N/A	CNS, CV	R
21^[32]^	N/A	N/A	N/A	Amitriptyline Zopiclone Venlafaxine Alcohol	4.8 g N/A N/A N/A	4.92 .8 3.20 –	Prolonged QTc	3	20% LE	N/A	N/A	CV	R
22^[33]^	F	Young	Depression	Bupropion Sertraline	N/A	3.6 5.51	525 ms	N/A	20% LE	N/A	N/A	CV	R
23^[34]^	F	53	Depression	Venlafaxine Amitriptyline Citalopram	N/A	3.2 4.92 3.76	Prolonged QT	3	20% Intralipid	N/A	N/A	CV	CNS sequelae
24^[35]^	F	35	N/A	Quetiapine	36 g	2.81	558 ms	N/A	20% LE	440 ms	N/A	CV	R
25^[35]^	F	64	Bipolar disorder CKD stage III COPD Hypertension	Quetiapine	8.7 g	2.81	N/A	N/A	20% LE	N/A	N/A	CV	CNS sequelae
26^[36]^	F	25	N/A	Amitriptyline	2.5 g	4.92	475 ms	10	20% LE	400 ms	N/A	CNS, CV	R
27^[37]^	F	19	Depression	Venlafaxine	18 g	3.2	N/A	N/A	20% LE	N/A	N/A	CNS, CV	R
28^[38]^	N/A	45	N/A	Amitriptyline	50 mg	4.92	589 ms	coma	20% LE	503 ms	N/A	CV	R
29^[39]^	M	18	Depression ADHD	Amitriptyline Venlafaxine	N/A	4.92 3.2	410 ms	N/A	20% LE	N/A	N/A	N/A	R
30^[40]^	F	28	N/A	Amitriptyline	5 g	4.92	N/A	N/A	20% LE	N/A	N/A	CV	R
31^[41]^	F	24	Depression	Chlorpromazine Mirtazapine	3000 mg 990 mg	5.41 2.9	N/A	3	20% Intralipid	N/A	N/A	CV	R
32^[42]^	F	51	N/A	Amitriptyline	925 mg	4.92	N/A	10	20% LE	N/A	14	CNS, CV	R
33^[42]^	F	24	N/A	Amitriptyline	875 mg	4.92	N/A	7	20% LE	N/A	9	CNS, CV	R
34^[42]^	F	32	N/A	Metoprolol	475 mg	2.15	N/A	15	20% LE	N/A	N/A	CV	R
35^[42]^	F	32	N/A	Fluoxetine Alprazolam Nifedipine	N/A	4.05 2.12 2.2	N/A	8	20% LE	N/A	8	CNS, CV	R
36^[42]^	M	28	N/A	Quetiapine	2400 mg	2.81	N/A	13	20% LE	N/A	15	CNS, CV	R
37^[42]^	F	18	Epilepsy	Lamotrigine Sertraline	N/A	2.57 5.51	N/A	12	20% LE	N/A	15	CNS	R
38^[42]^	M	17	N/A	Bonsai	N/A	N/A	N/A	9	20% LE	N/A	14	CNS, CV	R
39^[42]^	F	24	N/A	Amitriptyline	520 mg	4.92	N/A	12	20% LE	N/A	14	CNS, CV	R
40^[42]^	F	18	N/A	Amitriptyline α-lipoic acid	N/A	4.92 –	N/A	8	20% LE	N/A	N/A	N	D
41^[42]^	F	23	N/A	Amitriptyline	N/A	4.92	N/A	8	20% LE	N/A	13	CNS	N/A
42^[43]^	M	44	N/A	Amitriptyline	2.25 g	4.92	509 ms	N/A	20% LE	379 ms	N/A	CV	R
43^[44]^	F	29	N/A	Quetiapine Ibuprofen Escitalopram Amoxicillin	9 g 4 g 280 mg 5 g	2.81 3.97 3.74 .87	N/A	2T	20% LE	N/A	N/A	CNS	R
44^[45]^	M	50	N/A	Trazodone Cyclobenzaprine Doxepin	N/A	2.68 5.2 4.29	N/A	N/A	20% LE	N/A	N/A	CNS, CV	R
45^[46]^	F	20M	N/A	Dosulepin	450 mg	4.49	N/A	N/A	20% Intralipid	N/A	N/A	CV	R
46^[47]^	M	4	Epilepsy	Olanzapine	N/A	4.09	N/A	N/A	20% Intralipid	N/A	N/A	CNS, CV	R
47^[48]^	F	39	N/A	Olanzapine	100 mg	4.09	N/A	7	20% LE	N/A	N/A	CNS	R
48^[49]^	M	50	Bipolar disorder Type II DM	Lamotrigine	3.5 g	2.57	521 ms	7	20% LE	455 ms	N/A	CNS, CV	R
49^[50]^	F	44	Depression	Diazepam Lamotrigine Venlafaxine	200 mg 20 g 4.5 g	2.82 2.57 3.2	N/A	6	20% LE	N/A	N/A	CNS	R
50^[51]^	M	52	Depression Alcoholic liver disease Osteoporosis Exocrine pancreatic insufficiency Peptic ulcer disease B12 deficiency	Amitriptyline Liraglutide	N/A 36 mg	4.92 –	N/A	3	20% Intralipid	N/A	N/A	CV	R
51^[52]^	M	49	Depression	Trazodone	5000 mg	2.68	489 ms	11	20% Intralipid	N/A	N/A	CNS	R
52^[53]^	M	21	N/A	Amitriptyline	N/A	4.92	N/A	3	20% LE	N/A	10	CNS, CV	CNS sequelae
53^[54]^	M	55	Depression	Zopiclone Venlafaxine	N/A 1.8 g	.8 3.2	Normal	3	20% Intralipid	N/A	N/A	CNS	R
54^[55]^	F	30	N/A	Olanzapine	250 mg	4.09	Normal	9	20% LE	N/A	N/A	CNS	R
55^[56]^	M	22	N/A	Cyclobenzaprine	N/A	5.2	N/A	3	20% LE	N/A	N/A	CV	R
56^[57]^	M	61	Bipolar disorder Depression	Quetiapine Sertraline	4.3 g 3.1 g	2.81 5.51	Normal	3	20% Intralipid	N/A	15	CNS	R
57^[58]^	M	33	schizoaffective disorder	Quetiapine Venlafaxine	12 g 4.5 g	2.81 3.2	510 ms	4	20% Intralipid	N/A	N/A	CNS, CV	R
58^[59]^	M	48	ADHD Migraine Hypertension Cervical radiculopathy	Amitriptyline	N/A	4.92	N/A	N/A	20% LE	N/A	N/A	CNS, CV	R
59^[60]^	F	53	Depression Bipolar disorder ADHD Chronic pain syndrome	Clonidine Fluoxetine Bupropion gabapentin Quetiapine	6 g 1.2 g 13.5 g 9 g 6 g	1.59 4.05 3.6 1.25 2.81	N/A	N/A	20% LE	N/A	N/A	CNS, CV	R
60^[61]^	M	20	Intellectual disability ADHD	Olanzapine	840 mg	4.09	Normal	5	Clinoleic	Normal	N/A	CNS	R

ADHD = attention deficit hyperactivity disorder, CAD = coronary artery disease, CHF = congestive heart failure, CKD = chronic kidney disease, CNS = central nervous system, COPD = chronic obstructive pulmonary disease, CV = cardiovascular, D = death, DM = diabetes mellitus, F = female, GCS = Glasgow Coma Scale, IHD = ischemic heart disease, LE = lipid emulsion, Log *P* = log (octanol/water partition coefficient), M = male, N = none, N/A = not available, QT_C_ = corrected QT interval, R = recovery, T = intubation, WNL = within normal limits.

### 3.2. Log *P*, QTc, and GCS score

**Figure 2.**
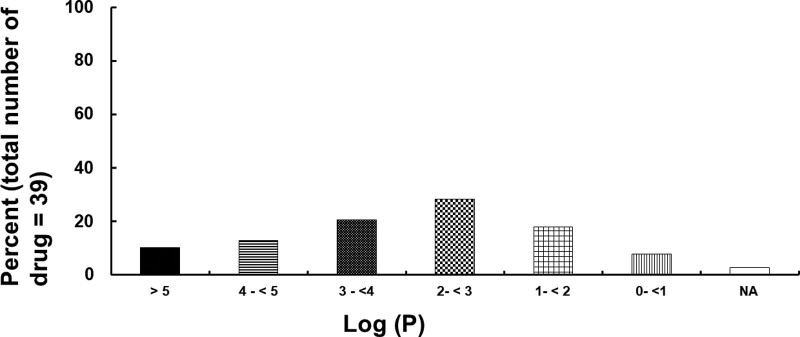
Distribution of lipid solubility (log *P*: log [octanol/water partition coefficient]) of neuropsychiatric drugs (total number of drugs: 39) that caused toxicity in patients undergoing lipid emulsion treatment. N/A = not available.

**Figure 3.**
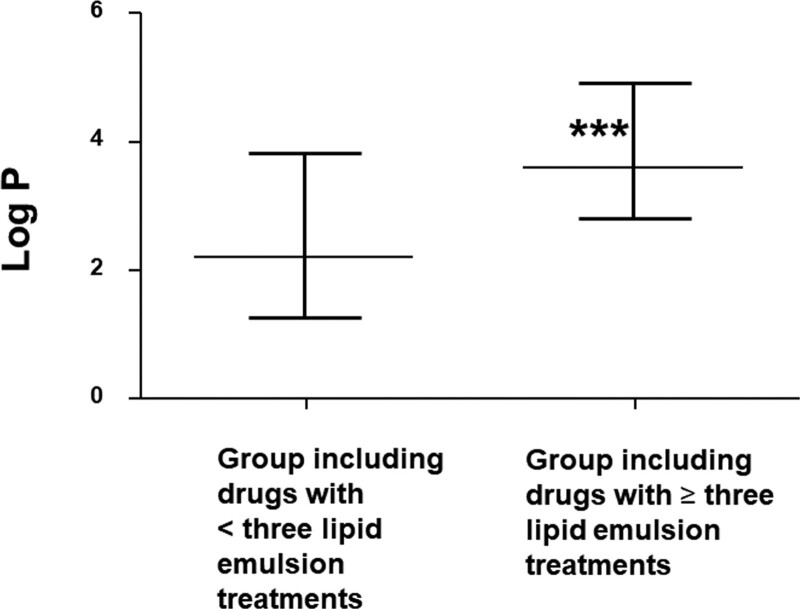
Comparison of lipid solubility (log *P*: log [octanol/water partition coefficient]) for the groups (including duplicates) that required more than or less than 3 lipid emulsion treatments; the total numbers of drugs in each group were 75 and 33, respectively. Data are shown as the median ± interquartile range (25–75%); ***P < .001 vs group which required less than 3 lipid emulsion treatments.

**Figure 4.**
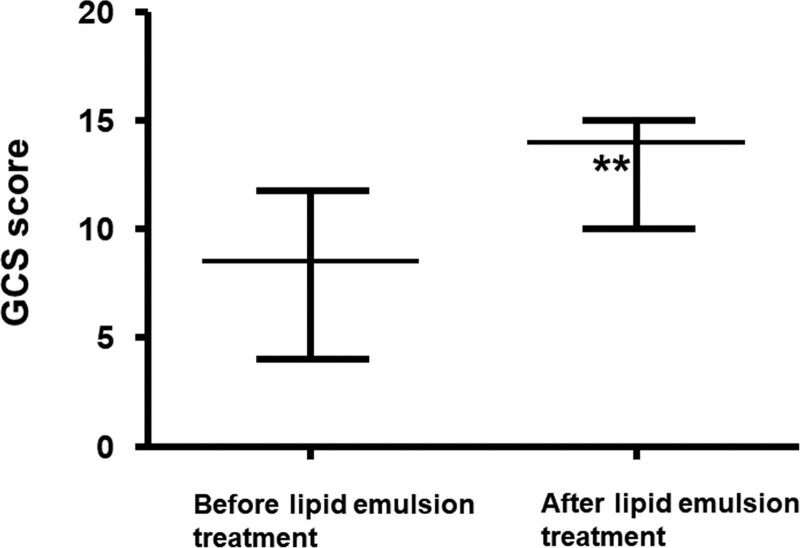
Effect of lipid emulsion on the GCS scores in patients (n = 12) undergoing lipid emulsion treatment for neuropsychiatric drug toxicity. Data are shown as the median ± interquartile range (25–75%); n indicates the number of patients and **P = .0037 vs before lipid emulsion treatment. GCS = Glasgow Coma Scale.

**Figure 5.**
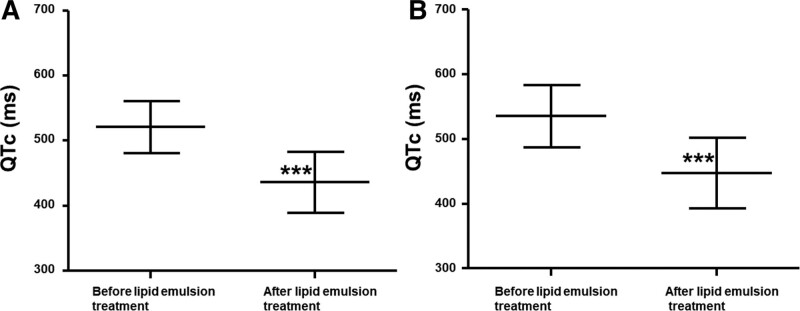
Effect of lipid emulsion on the prolonged corrected QTc interval (QTc) in patients undergoing lipid emulsion treatment for drug toxicity related to a single neuropsychiatric drug (A, n = 11) or single and multiple drugs (B, n = 16). Data are shown as the mean ± standard deviation; n indicates the number of patients. ***P < .001 vs before lipid emulsion treatment.

Toxicity due to a single drug or by multiple drugs occurred in 33 (55%) and 27 (45%) patients, respectively (Table 1). The distribution of the lipid solubility (log *P*) of neuropsychiatric drugs is shown in Figure 2. Of 39 drugs, 11 (28.21%) had a log *P* ≥ 2 to <3, while 28 (71.79%) had a log *P* of >2, and were considered highly lipid soluble.^[62]^ In addition, of 39 drugs, 7 (17.94%) and 3 (7.69%) had a log *P* of ≥1 to <2 and <1, respectively, and were considered less lipid soluble. In addition, the log *P* (median [interquartile range: 25–75%]: 3.6 [2.81–4.92]) of the group which required more than 3 lipid emulsion treatments was higher than that (log *P*: 2.2 [1.28–3.82]) of the group which required less than 3 lipid emulsion treatments (*P* < .001; Fig. 3). Lipid emulsion significantly increased the GCS from 8.5 [4–11.75] (median [interquartile range: 25–75%]) to 14 [10–15] in 12 patients (Fig. 4, *P* = .0037). Lipid emulsion also significantly shortened single drug-induced prolonged QTc (QTc: mean ± standard deviation [SD]; before lipid emulsion treatment = 520.8 ± 39.5 ms, after lipid emulsion treatment = 436.1 ± 46.8 ms) in 11 patients (Fig. 5A, *P *< .001). It also significantly shortened single and multiple drug-induced prolonged QTc from 535 ± 48 to 447 ± 54 ms (Fig. 5B, *P* < .001) in 16 patients.

### 3.3. Lipid emulsion treatment

Intralipid, which contains 100% long-chain fatty acids, was the most commonly used lipid emulsion (17 patients; 28.3%) for the treatment of neuropsychiatric drug toxicity (Table 1). However, many case reports (38 patients, 63.33%) described only 20% lipid emulsions. The method of administration of lipid emulsions in neuropsychiatric drug toxicity was as follows (Table S1, Supplemental Digital Content, http://links.lww.com/MD/L976): bolus administration followed by continuous infusion (40 patients, 66.6%), bolus administration only (8 patients, 13.38%), and continuous infusion only (7 patients, 11.6%). Among patients who underwent most commonly employed administration method (bolus administration followed by continuous infusion; 40 patients), a 1.5 mL/kg bolus of 20% lipid emulsion followed by 0.25 mL/kg/min continuous infusion of 20% lipid emulsion was most frequently used (12/40, 30%). Patients also commonly received a 100 mL bolus followed by 0.5 mL/kg/min continuous infusion of the lipid emulsion (10/40, 25%) (Table S1, Supplemental Digital Content, http://links.lww.com/MD/L976). The main rationale to administer lipid emulsion as an adjuvant drug for toxicity caused by neuropsychiatric drugs was as follows (Table 1): hemodynamic depression intractable to supportive treatment (53 patients, 88.3%) > lipophilic drugs (5 patients, 8.3%; Case No.: 43, 46, 47, 53, 56) > no spontaneous breathing and alertness (1 patient, 1.6%; Case No.: 60) or suspected overdose (1 patient, 1.6%, Case No.: 51; Table 1).

### 3.4. Symptom improvement and side effects

The frequency of symptom improvement after lipid emulsion administration was as follows (Table 1): cardiovascular and central nervous system symptoms (24 patients, 40%), cardiovascular symptoms alone (23 patients, 38.3%), and central nervous system symptoms alone (10 patients, 16.7%). However, lipid emulsion did not improve symptoms in 1 patient (1.61%; Table 1). Lipid emulsion generally improved symptoms rapidly (within 5 minutes, 10–30 minutes, and 30–60 minutes after lipid emulsion administration in 14 (23.3%), 2 (3.3%), 10 (16.7%), and 8 (13.3%) patients, respectively) (Table S1, Supplemental Digital Content, http://links.lww.com/MD/L976). The total volume of lipid emulsion administered was as follows (Table S1, Supplemental Digital Content, http://links.lww.com/MD/L976): ≤500 mL (21 patients, 35%), >3000 to ≤4000 mL (7 patients, 11.7%), >500 to ≤1000 mL (7 patients, 11.7%), >1000 to ≤2000 mL (3 patients, 5%), >4000 to ≤5000 mL (1 patient, 1.6%), or >5000 mL (1 patient, 1.6%). The administration of lipid emulsion as an adjuvant drug resulted in full recovery from neuropsychiatric drug toxicity in 52 patients (86.7%; Table 1). In addition, 5 patients recovered with central nervous sequelae, and another recovered but with sustained prolonged QTc (Table 1). One patient died despite receiving lipid emulsion treatment (Table 1). Residual central nervous system sequelae after lipid emulsion treatment for drug toxicity caused by an overdose of multiple drugs (bupropion and lamotrigine or venlafaxine, amitriptyline, and citalopram) or a single drug (lamotrigine, quetiapine, and amitriptyline) included slight tremors, altered mental status, neurocognitive dysfunction, and lack of alertness (Case No.: 15, 19, 23, 25, 52; Table 1). The side effects of lipid emulsion resuscitation included hyperlipidemia, hypertriglyceridemia, adult respiratory distress syndrome, lung infiltration, pancreatitis, increased amylase and lipase levels, ground-glass opacities of the lungs, and increased aspartate aminotransferase and alanine aminotransferase levels.

### 3.5. Posttreatment symptom recurrence

In some cases, symptoms initially improved following the administration of lipid emulsion but later deteriorated. These later-onset symptoms included altered mental status, a widened QRS interval, hypotension, pulseless wide-complex tachycardia, apnea, pupil dilation, decreased GCS scores, tachycardia, and somnolence. The drugs associated with these recurrent symptoms included bupropion, amitriptyline, fluoxetine, escitalopram, olanzapine, quetiapine, pregabalin, gabapentin, and propranolol.

## 4. Discussion

The results of this review show that administering lipid emulsion as an adjuvant drug shortens QTc prolongation and improves GCS scores in patients with intractable drug toxicity caused by toxic doses of lipid-soluble neuropsychiatric drugs, leading to improved recovery.

Phase 3 of the cardiac action potential involves inactivation of inward calcium currents and activation of rapid outward potassium currents with slow potassium currents and inward rectifying potassium currents.^[6]^ QT prolongation may occur when the outward potassium current is decreased or inward calcium or sodium currents is increased during phase 3 of the cardiac action potential.^[6]^ Antidepressants (e.g., amitriptyline, fluoxetine, imipramine, and doxepine) and antipsychotics (e.g., quetiapine, haloperidol, chloropromazine, amisulpride, and risperidone) cause QT prolongation, leading to ventricular fibrillation and cardiac arrest.^[6]^ Local anesthetic inhibits human ether-a-go-go-related gene cardiac potassium channels, which codes for rapid delayed rectified potassium channels.^[63]^ Bupivacaine (log *P*: 3.41) is a highly lipid-soluble local anesthetic which induces QTc prolongation, and lipid emulsion reversed bupivacaine-related increased Tpeak-to-Tend intervals (transmural dispersion).^[64]^ A randomized controlled study reported that lipid emulsion reversed clozapine (log *P*: 2.41) toxicity-related QTc prolongation.^[7]^ In addition, lipid emulsion reversed QTc prolongation and inhibited myocardial cell death caused by amitriptyline toxicity in rats.^[65]^ Similar to these previous reports,^[7,64,65]^ lipid emulsion ameliorated QTc prolongation caused by toxic doses of neuropsychiatric drugs, including amitriptyline (log *P*: 4.92), trazodone (log *P*: 2.68), desulepin (log *P*: 4.49), haloperidol (log *P*: 4.3), lamotrigine (log *P*: 2.57), and quetiapine (log *P*: 2.81). The above-mentioned neuropsychiatric drugs (log *P*: 3.55 [2.65–4.59]) are highly lipid-soluble (log *P*: >2; Table 1). In addition, lipid emulsion alone has positive inotropic and lusitropic effects.^[66,67]^ Considering previous reports, this lipid emulsion-mediated reversal of QTc prolongation and positive inotropy may contribute to the reversal of neuropsychiatric drug toxicity-induced cardiac depression.^[1,6,7,63–67]^ However, retrospective analyses of case reports using lipid emulsion as an adjuvant drug have suggested that lipid emulsion does not significantly improve QTc prolongation caused by antihistamine diphenhydramine toxicity.^[68]^ This difference may reflect the small sample size of the lipid emulsion group in the previous study, and differences in experimental methods (with vs without a control group).

Local anesthetic-related systemic toxicity usually involves the central nervous system, followed by symptoms associated with the cardiovascular system.^[1]^ Early lipid emulsion administration in patients with bupivacaine- or ropivacaine-induced central nervous system symptoms prior to the onset of cardiac symptoms treated perioral numbness, restlessness, agitation, dizziness, and dysarthria.^[69,70]^ In addition, a randomized controlled study suggested that lipid emulsion shortened the recovery time from isoflurane anesthesia, leading to improved recovery quality.^[71,72]^ An animal study also corroborated that lipid emulsion shortened the time from isoflurane anesthesia to recovery, and decreased the proportion of the delta-band in the electroencephalogram during anesthesia, which appears to be mediated by lipid emulsion-induced reduction of isoflurane concentrations.^[9]^ Lipid emulsion and supportive treatment improved the GCS of patients with drug toxicity due to nonlocal anesthetic drugs or clozapine alone.^[7,8]^ Consistent with previous reports,^[7,8,72]^ in our analysis, lipid emulsion reversed the decreased GCS score related to overdoses of the following: amitriptyline (log *P*: 4.92), trazodone (log *P*: 2.68), quetiapine (log *P*: 2.81), citalopram (log *P*: 3.76), bromazepam (log *P*: 2.05), fluoxetine (log *P*: 4.05), alprazolam (log *P*: 2.12), lamotrigine (log *P*: 2.57), and sertraline (log *P*: 5.51), which are all highly-soluble (log *P*: 3.38 ± 1.24). Moreover, lipid emulsion treatment in intravenous amitriptyline toxicity increased arterial plasma amitriptyline concentrations but reduced brain amitriptyline concentrations, suggesting lipid emulsion-induced sequestration of amitriptyline from the brain to the blood.^[73]^ Thus, considering these previous laboratory reports, the increase in GCS after lipid emulsion administration may be associated with increased partitioning of highly lipid-soluble neuropsychiatric drugs into the blood from the brain.^[9,73]^ However, further studies are needed to examine the detailed mechanism responsible for this phenomenon.

The magnitude of lipid emulsion-induced decreases in serum drug concentrations was reported to be strongly correlated with the lipid solubility of drugs.^[74]^ Lipid emulsion inhibited the decreased blood pressure caused by toxic doses of verapamil, which has high lipid solubility (log *P*: 3.79), but had no effect on that caused by diltiazem, which has relatively low lipid solubility (log *P*: 2.8).^[75]^ Moreover, lipid emulsion inhibited the decreased cell viability caused by verapamil toxicity in rat cardiomyoblasts more than that induced by diltiazem toxicity.^[76]^ Lipid emulsion inhibited propranolol-induced hypotension, which is highly lipid-soluble (log *P*: 3.48).^[77]^ However, it exerted no effect on metoprolol-related hypotension, which is less lipid-soluble (log *P*: 2.15).^[78]^ Taken together, these previous results suggest that lipid emulsion-mediated treatment is dependent on the lipid solubility of the offending drugs.^[74–78]^ Lipid emulsion resuscitation is believed to be mediated by a lipid shuttle which forms via binding of the lipid emulsion to the offending drugs, allowing for their redistribution.^[79]^ Intravenously-administered lipid emulsions generate a lipid phase component in the blood, which adsorbs lipid-soluble drugs from the heart and brain, which receive high blood flows.^[79]^ The drug-containing lipid emulsion is then delivered to the liver, muscle, and adipose tissue for detoxification and storage.^[79]^ When lipid emulsions were given 30 minutes after intravenous amitriptyline toxicity, amitriptyline concentrations in the heart and brain decreased while that in the arterial plasma increased, suggesting lipid emulsion-mediated partitioning from organs with high blood flow to the arterial blood.^[73]^ Consistent with previous reports, 71.79% (28/39) of drugs that underwent lipid emulsion treatment for neuropsychiatric drug toxicity were highly lipid-soluble (log *P*: >2).^[62]^ Moreover, the lipid solubility (log *P*) of the drugs for the group which required more than 3 lipid emulsion treatments were higher than that of the group which required less than 3 lipid emulsion treatments (Fig. 3). Taken together, these results suggest that highly lipid-soluble drugs which produce neuropsychiatric drug toxicity require more lipid emulsion treatments than less lipid-soluble drugs.^[74–78]^ Furthermore, as the log *P* of all drugs to produce improved QTc and GCS was more than 2, the high lipid solubility of these drugs may have contributed to the recovery of 96.66% (58/60) of patients from neuropsychiatric drug toxicity by shortening QTc prolongation and increasing GCS score. Bolus administration of 1.5 mL/kg lipid emulsion (20%) followed by 0.25 mL/kg/min continuous infusion of 20% lipid emulsion was most frequently employed (12/60, 20%), which is the recommended dosing regimen to treat local anesthetic-related systemic toxicity.^[1]^ However, local anesthetic systemic toxicity mostly occurs via inadvertently intravenous administration, whereas neuropsychiatric drug toxicity most occurs via oral administration. Thus, the toxicokinetics of drug toxicity due to toxic doses of local anesthetic and nonlocal anesthetic drugs are different. Furthermore, intravenous lipid emulsion administered 30 minutes after amitriptyline toxicity via orogastric administration increased blood amitriptyline concentrations, suggesting lipid emulsion-induced increased absorption from the gastrointestinal tract.^[80]^ Thus, further studies are needed to determine the optimal dosing regimen of lipid emulsion treatment for nonlocal anesthetic drug toxicity via oral administration. Studies have shown that 1% plasma triglyceride has both scavenging and positive inotropic and lusitropic effects.^[66,67,81]^ In addition, the maximum Intralipid (10%) clearing capacity (K_1_) has been reported to be 110 ± 4 μM/L/min.^[82]^ One previous report suggested the following dosing regimen for 20% lipid emulsion treatment for nonlocal anesthetic drug toxicity via oral administration, which produced a 1% plasma triglyceride concentration: 1.5 mL/kg bolus administration, then 0.25 mL/kg/min continuous infusion for 3 minutes, followed by 0.025 mL/kg/min continuous infusion.^[66,67,80–83]^

Previous studies have suggested that lipid emulsion treatment improves various symptoms caused by nonlocal anesthetic drug toxicity in the following order of frequency: symptoms of the cardiovascular system alone > symptoms of the central nervous system alone > symptoms associated with both the cardiovascular and central nervous systems.^[2,3]^ However, in the present study, lipid emulsion treatment most commonly improved symptoms associated with the cardiovascular and central nervous systems, followed by the cardiovascular system alone, followed by the central nervous system alone. This difference may be due to differences in the involved drugs.^[2,3]^ Consistent with a previous report, the main reason for prescribing lipid emulsion treatment in neuropsychiatric drug toxicity was hemodynamic depression (53/60, 88.3%) that was resistant to supportive treatment.^[2]^ A meta-analysis of lipid emulsion treatment for nonlocal anesthetic-induced toxicity reported that lipid emulsion treatment reduced the odds ratio of mortality to 0.43.^[84]^ After lipid emulsion treatment, most patients (52/60, 86.6%) fully recovered from neuropsychiatric drug toxicity which was intractable to supportive treatment. Similar to previous reports, lipid emulsion treatment produced the following side effects: lipemia, adult respiratory distress syndrome, pancreatitis, and hypertriglyceridemia.^[85,86]^ On the other hand, these side effects may be partially due to underlying conditions rather than lipid emulsion treatment itself (protopathic bias).^[87]^ Consistent with previous reports, Intralipid was commonly used for the treatment of neuropsychiatric drug toxicity.^[2,3]^ However, the availability of Intralipid is limited because linoleic acid, which is the main long-chain fatty acid in Intralipid, produces pro-inflammatory mediators and induces lipid perioxidation.^[88]^ However, alternative lipid emulsion preparations can be used in the treatment of cardiovascular depression related to local anesthetic systemic toxicity, such as Lipofundin MCT/LCT, SMOFlipid, and Clinoelic.^[89]^ Thus, other alternative lipid emulsions such as Lipofundin MCT/LCT, SMOFlipid, and Clinoelic, can be used as adjuvant drugs with supportive treatment in treating nonlocal anesthetic drug toxicity involving critical cardiovascular depression. In the current analysis, lipid emulsion administration led to full recovery from some cases of neuropsychiatric drug toxicity. However, other patients with toxicity induced by the same drug did not fully recover. These contrasting results may be due to the dosage of the neuropsychiatric drug, predisposing factors, lipid emulsion dosage, timing of lipid emulsion administration, and additional supportive treatments used.

### 4.1. Limitations of this study

We examined the effect of lipid emulsion as an adjuvant drug on toxicity related to various neuropsychiatric drugs. However, examining the effect of lipid emulsions on drug toxicity caused by a single antidepressant or antipsychotic is more appropriate for reaching clear conclusions. Second, negative results (no beneficial effect of supportive treatment plus lipid emulsion compared with supportive treatment alone) regarding lipid emulsion treatment of neuropsychiatric drug toxicity are relatively more difficult to publish compared with positive results, which skews the results of case-based analysis. Third, this analysis only used case reports of patients treated with both lipid emulsion and supportive treatment, and an analysis comparing lipid emulsion and supportive treatment with supportive treatment alone is needed to confirm our findings. Despite these limitations, our study results hold value as it is impossible to perform a completely randomized clinical study to examine the effects of lipid emulsions on drug toxicity in humans for various reasons, including ethical issues. In addition, several case reports regarding lipid emulsion treatment for neuropsychiatric drug toxicity are available. The present study is a comprehensive systemic review of case reports regarding lipid emulsion treatment for neuropsychiatric drug toxicity, with a particular focus on GCS scores, QTc, and the lipophilicity of the offending drugs. Thus, this analysis may be helpful in guiding lipid emulsion treatment for neuropsychiatric drug toxicity. Further analysis using additional case reports accumulated through the lipid emulsion resuscitation registry is needed to reach clearer conclusions.

## 5. Conclusions

Taken together, lipid emulsion treatment as an adjuvant drug induces the reversal of decreased GCS scores and QTc prolongation caused by toxic doses of highly lipid-soluble neuropsychiatric drugs (amitriptyline, trazodone, quetiapine, lamotrigine, and citalopram) which are resistant to supportive treatment, leading to improved recovery from neuropsychiatric drug toxicity. However, more studies on large cohorts and analyses using many case reports are needed to clarify the effects of lipid emulsion resuscitation on neuropsychiatric drug toxicity and its underlying mechanisms.

## Author contributions

**Conceptualization:** Yeran Hwang, Ju-Tae Sohn.

**Data curation:** Yeran Hwang.

**Formal analysis:** Yeran Hwang, Ju-Tae Sohn.

**Funding acquisition:** Ju-Tae Sohn.

**Investigation:** Yeran Hwang, Ju-Tae Sohn.

**Methodology:** Yeran Hwang, Ju-Tae Sohn.

**Project administration:** Ju-Tae Sohn.

**Resources:** Yeran Hwang, Ju-Tae Sohn.

**Software:** Yeran Hwang, Ju-Tae Sohn.

**Supervision:** Ju-Tae Sohn.

**Validation:** Yeran Hwang, Ju-Tae Sohn.

**Visualization:** Ju-Tae Sohn.

**Writing – original draft:** Ju-Tae Sohn.

**Writing – review & editing:** Yeran Hwang, Ju-Tae Sohn.

## Supplementary Material


