# Supplementary material for: Effect of lipid emulsion on neuropsychiatric drug-induced toxicity: A narrative review
Source: Medicine (Baltimore). 2024 Mar 15;103(11):e37612. doi: 10.1097/MD.0000000000037612 (PMC10939703; doi:10.1097/MD.0000000000037612)
Supplement: Supplementary file 1 [file medi-103-e37612-s001.docx]

Supplementary table 1. Lipid emulsion treatment for toxicity caused by neuropsychiatric drugs (e.g., antidepressants, antipsychotics, benzodiazepines, and anticonvulsants)

| Case No. | Sex | Age | Underlying  disease | Drug | Dosage | Log P | Bolus  Administration  of LE | Continuous  Infusion of LE | Total Dose | Time From LE  Administration to  Symptom Improvement |
| --- | --- | --- | --- | --- | --- | --- | --- | --- | --- | --- |
| 1^14^ | F | 28 | Depression | Amitriptyline | 3.7 g | 4.92 | 1.5 mL/kg | 0.25 mL/kg/min | 540 ml | 10 min |
| 2^15^ | F | 77 | Depression | Trazodone | 4.5 g | 2.68 | 1.5 mL/kg | 0.25 mL/kg/min | N/A | Immediately |
| 3^16^ | F | 15 | N | Bupropion | 1,650 to  9,000 mg | 3.6 | N | 1.5 ml/kg | N/A | 30 min |
| 4^17^ | N/A | 25 | Depression | Amitriptyline  Quetiapine | 5.5 g  10 g | 4.92  2.81 | N | 250 ml | 250 ml | 30 min |
| 5^18^ | F | 19 | N | Quetiapine  Citalopram  Bromazepam | 6000 mg  400 mg  45 mg | 2.81  3.76  2.05 | 100 ml | 200 ml/2hr | 300 ml | N/A |
| 6^19^ | F | 14 | N/A | Bupropion  Hydroxyzine  Citalopram | 9 g  N/A  N/A | 3.6  2.36  3.76 | 1st : 100ml  2nd : 100ml | 1st : 0.25 ml/kg/min  2nd : 0.25ml/kg/min  3rd : 0.25ml/kg/min | 4700 ml | N/A |
| 7^20^ | M | 21 | Depression | Citalopram  Clonazepam  Olanzapine | 11.6 g  5 mg  600 mg | 3.76  2.41  3.0 | N | 0.005 ml/kg/min  (21 ml/hr) | 375 ml | 24 hr |
| 8^21^ | F | 42 | Schizoaffective disorder | Quetiapine | 24 g | 2.81 | 170 ml | 500 ml/1hr | 670 ml | 3 hr |
| 9^22^ | F | 34 | N/A | Amitriptyline  Citalopram | 5600 mg  2400 mg | 4.92  3.76 | 1.5 ml/kg | 0.25 ml/kg/min | 1000 ml | immediately |
| 10^23^ | F | 13 | N/A | Amitriptyline  Nortriptyline | N/A | 4.92  3.9 | 1st : 1.5 mg/kg  2nd :1.5mg/kg | 0.25 mg/kg/min | N/A | immediately |
| 11^24^ | F | 25 | Anorexia  Depression | Amitriptyline Fluoxetine  Escitalopram  Olanzapine  Quetiapine  Gabapentin | N/A | 4.92  4.05  3.74  3.0  2.81  1.25 | 1st : 150 ml  2nd : 40ml | 1st : 16 ml/hr  2nd : 16ml/hr | 814 ml | 36 hr |
| 12^25^ | M | 51 | Depression  IHD | Amitriptyline  Quetiapine  Citalopram  Metoprolol  Quinapril  Aspirin | 3250 mg  N/A  N/A  N/A  N/A  N/A | 4.92  2.81  3.76  2.15  0.86  1.18 | 100 ml | 400 ml/30min | 500 ml | Immediately |
| 13^26^ | F | 36 | N/A | Dothiepin | 2250 mg | 4.49 | 1.5 ml/kg | 0.25 ml/kg/min | 500 ml | Immediately |
| 14^27^ | F | 36 | Schizophrenia | Dosulepin | 5.25 g | 4.49 | 1.5 mL/kg | 400 ml/20min | 500 ml | 15 min |
| 15^5^ | F | 17 | Bipolar disorder | Bupropion  Lamotrigine | 4 g  7.95 g | 3.6  2.57 | 100 ml | N | 100 ml | 15 min |
| 16^28^ | F | 45 | Anxiety  Depression  Hypertension | Haloperidol | 5 g | 4.3 | 250 ml | N | 250 ml | 2 min |
| 17^29^ | M | 54 | Anxiety  Depression  CAD  CHF  COPD  type II DM | Trazodone | 100 mg | 2.68 | 1 ml/kg | 0.25 mL/kg/hr | N/A | 2 min |
| 18^30^ | F | 45 | N/A | Amisulpride  Diazepam  Valsartan  Aripiprazole  Paliperidone | 28 g  250 mg  2,240 mg  45 mg  21 mg | 1.06  2.82  1.499  5.30  1.8 | N | 100ml/hr | N/A | 1 hr |
| 19^31^ | M | 36 | Bipolar disorder | Lamotrigine | 13.5 g | 2.57 | 1.5 ml/kg | 0.5 mL/kg/min | N/A | Immediately |
| 20^32^ | F | 25 | N/A | Amitriptyline  Propranolol  Pregabalin | N/A | 4.92  3.48  1.3 | 1st : 1.5 ml/kg  2nd : 1.5ml/kg | 1st : 75 ml/hr  2nd : 25ml/hr | 636 ml | Immediately |
| 21^33^ | N/A | N/A | N/A | Amitriptyline  Zopiclone  Venlafaxine  Alcohol | 4.8 g  N/A  N/A  N/A | 4.92  0.8  3.20  - | N/A | N/A | N/A | N/A |
| 22^34^ | F | young | Depression | Bupropion  Sertraline | N/A | 3.6  5.51 | 1.5 mL/kg | 1.5 mL/kg/1hr | 140 ml | immediately |
| 23^35^ | F | 53 | Depression | Venlafaxine  Amitriptyline  Citalopram | N/A | 3.2  4.92  3.76 | N/A | N/A | 500 ml | immediately |
| 24^36^ | F | 35 | N/A | Quetiapine | 36 g | 2.81 | 1.5 mL/kg | 0.25 mL/kg/min | 2000 ml | N/A |
| 25^36^ | F | 64 | Bipolar disorder  CKD stage III  COPD  Hypertension | Quetiapine | 8.7 g | 2.81 | N/A | 0.25 mL/kg/min | N/A | 1 hr |
| 26^37^ | F | 25 | N/A | Amitriptyline | 2.5 g | 4.92 | N/A | 500 ml/2hr | 500 ml | N/A |
| 27^38^ | F | 19 | Depression | Venlafaxine | 18 g | 3.2 | 2.5 mL/kg | 0.25 mL/kg/min | 1050 ml | N/A |
| 28^39^ | N/A | 45 | N/A | Amitriptyline | 50 mg | 4.92 | 100 ml | 400 ml/30min | 500 ml | Immediately |
| 29^40^ | M | 18 | Depression  ADHD | Amitriptyline  Venlafaxine | N/A | 4.92  3.2 | N/A | N/A | N/A | N/A |
| 30^41^ | F | 28 | N/A | Amitriptyline | 5 g | 4.92 | N/A | N/A | N/A | N/A |
| 31^42^ | F | 24 | Depression | Chlorpromazine  Mirtazapine | 3000 mg  990 mg | 5.41  2.9 | 1st : 100 ml  2nd : 100ml | N/A | 200 ml | 20 min |
| 32^43^ | F | 51 | N/A | Amitriptyline | 925 mg | 4.92 | 100 ml | 0.5 ml/kg/min | N/A | 1 hr |
| 33^43^ | F | 24 | N/A | Amitriptyline | 875 mg | 4.92 | 100 ml | 0.5 ml/kg/min | 3400 ml | 45 min |
| 34^43^ | F | 32 | N/A | Metoprolol | 475 mg | 2.15 | 100 ml | 0.5 ml/kg/min | 3100 ml | 2 hr |
| 35^43^ | F | 32 | N/A | Fluoxetine Alprazolam  Nifedipine | N/A | 4.05  2.12  2.2 | 100 ml | 0.5 ml/kg/min | 3700 ml | 2 hr |
| 36^43^ | M | 28 | N/A | Quetiapine | 2400 mg | 2.81 | 100 ml | 0.5 ml/kg/min | 3580 ml | 2 hr |
| 37^43^ | F | 18 | Epilepsy | Lamotrigine  Sertraline | N/A | 2.57  5.51 | 100 ml | 0.5 ml/kg/min | 3100 ml | 1 hr |
| 38^43^ | M | 17 | N/A | Bonsai | N/A | N/A | 100 ml | 0.5 ml/kg/min | 4000 ml | 2 hr |
| 39^43^ | F | 24 | N/A | Amitriptyline | 520 mg | 4.92 | 100 ml | 0.5 ml/kg/min | 3400 ml | 1 hr |
| 40^43^ | F | 18 | N/A | Amitriptyline  α-lipoic acid | N/A | 4.92  - | 100 ml | 0.5 ml/kg/min | 500 ml | 20 min |
| 41^43^ | F | 23 | N/A | Amitriptyline | N/A | 4.92 | 100 ml | 0.5 ml/kg/min | 1750 ml | 1 hr |
| 42^44^ | M | 44 | N/A | Amitriptyline | 2.25 g | 4.92 | 250 ml | 1st : 100 ml/hr  2nd : 18ml/hr | 9562 ml | N/A |
| 43^45^ | F | 29 | N/A | Quetiapine  Ibuprofen  Escitalopram  Amoxicillin | 9 g  4 g  280 mg  5 g | 2.81  3.97  3.74  0.87 | 1st : 1.5 ml/kg  2nd : 1.5ml/kg | N | N/A | 30 min |
| 44^46^ | M | 50 | N/A | Trazodone  Cyclobenzaprine  Doxepin | N/A | 2.68  5.2  4.29 | 1.5 ml/kg | 0.25 mg/kg/min | N/A | 2 hr |
| 45^47^ | F | 20M | N/A | Dosulepin | 450 mg | 4.49 | 10 ml | 0.25 mL/kg/min | 160 ml | N/A |
| 46^48^ | M | 4 | Epilepsy | Olanzapine | N/A | 4.09 | 1.5 ml/kg | 0.25 ml/kg/min | N/A | 15 min |
| 47^49^ | F | 39 | N/A | Olanzapine | 100 mg | 4.09 | 100 ml | 100 ml/30min | 200 ml | immediately |
| 48^50^ | M | 50 | Bipolar disorder  Type II DM | Lamotrigine | 3.5 g | 2.57 | 1.5 mL/kg | 0.5 mL/kg/min | N/A | immediately |
| 49^51^ | F | 44 | Depression | Diazepam  Lamotrigine  Venlafaxine | 200 mg  20 g  4.5 g | 2.82  2.57  3.2 | 150 ml | N | 150 ml | immediately |
| 50^52^ | M | 52 | Depression  Alcoholic liver disease  Osteoporosis  Exocrine pancreatic insufficiency  Peptic ulcer disease  B12 deficiency | Amitriptyline  Liraglutide | N/A  36 mg | 4.92  - | 100 ml | N | 100 ml | N/A |
| 51^53^ | M | 49 | Depression | Trazodone | 5000 mg | 2.68 | 1.5 ml/kg | 0.25 mg/kg/min | N/A | 1 hr |
| 52^54^ | M | 21 | N/A | Amitriptyline | N/A | 4.92 | 40 mg | N/A | N/A | N/A |
| 53^55^ | M | 55 | Depression | Zopiclone  Venlafaxine | N/A  1.8 g | 0.8  3.2 | 1.5 ml/kg | 400 ml/40min | 500 ml | 30 min |
| 54^56^ | F | 30 | N/A | Olanzapine | 250 mg | 4.09 | 1.5 ml/kg | 0.25 ml/kg/min | N/A | 3 hr |
| 55^57^ | M | 22 | N/A | Cyclobenzaprine | N/A | 5.2 | 100 ml | N | N/A | N/A |
| 56^58^ | M | 61 | Bipolar disorder Depression | Quetiapine Sertraline | 4.3 g  3.1 g | 2.81  5.51 | 1.5 ml/kg | 6 ml/kg/hr | 500 ml | immediately |
| 57^59^ | M | 33 | schizoaffective disorder | Quetiapine  Venlafaxine | 12 g  4.5 g | 2.81  3.2 | 1.5 ml/kg | 0.1 ml/kg/min | 600 ml | N/A |
| 58^60^ | M | 48 | ADHD  Migraine  Hypertension  Cervical radiculopathy | Amitriptyline | N/A | 4.92 | 1.5 ml/kg | 0.25 ml/kg/min | N/A | N/A |
| 59^61^ | F | 53 | Depression  Bipolar disorder  ADHD  Chronic pain syndrome | Clonidine  Fluoxetine  Bupropion  gabapentin  Quetiapine | 6 g  1.2 g  13.5 g  9 g  6 g | 1.59  4.05  3.6  1.25  2.81 | N/A | N/A | N/A | N/A |
| 60^62^ | M | 20 | Intellectual disability  ADHD | Olanzapine | 840 mg | 4.09 | 1.5 ml/kg | 1.5 mL/min | 1000 ml | 6hr |

ADHD, Attention Deficit Hyperactivity Disorder; CAD, Coronary Artery Disease; CHF, Congestive Heart Failure; CKD, Chronic Kidney Disease; COPD, Chronic Obstructive Pulmonary Disease; DM, Diabetes Mellitus; F, Female; IHD, Ischemic Heart Disease; LE, Lipid Emulsion; Log P, log (octanol/water partition coefficient); M, Male; N, None; N/A, Not Available.
